# The subjectively perceived quality of postgraduate medical training in integrative medicine within the public healthcare systems of Germany and Switzerland: the example of anthroposophic hospitals

**DOI:** 10.1186/1472-6882-14-191

**Published:** 2014-06-16

**Authors:** Peter Heusser, Sabine Eberhard, Bettina Berger, Johannes Weinzirl, Pascale Orlow

**Affiliations:** 1 Gerhard Kienle Chair for Theory of Medicine, Integrative and Anthroposophic Medicine, Institute of Integrative Medicine, Witten/Herdecke University, Gerhard-Kienle-Weg 4, D-58313 Herdecke, Germany; 2 Institute for Environmental Decisions (IED) Consumer Behavior, Swiss Federal Institute of Technology (ETH), Zürich, Switzerland

**Keywords:** Postgraduate medical training, Residency training, Evaluation, Quality, Problems, Anthroposophic medicine, Integrative medicine

## Abstract

**Background:**

Integrative medicine (IM) integrates evidence-based Complementary and Alternative Medicine (CAM) with conventional medicine (CON). Medical schools offer basic CAM electives but in postgraduate medical training (PGMT) little has been done for the integration of CAM. An exception to this is anthroposophic medicine (AM), a western form of CAM based on CON, offering an individualized holistic IM approach. AM hospitals are part of the public healthcare systems in Germany and Switzerland and train AM in PGMT. We performed the first quality evaluation of the subjectively perceived quality of this PGMT.

**Methods:**

An anonymous full survey of all 214 trainers (TR) and 240 trainees (TE) in all 15 AM hospitals in Germany and Switzerland, using the ETHZ questionnaire for annual national PGMT assessments in Switzerland (CH) and Germany (D), complemented by a module for AM. Data analysis included Cronbach’s alpha to assess internal consistency questionnaire scales, 2-tailed Pearson correlation of specific quality dimensions of PGMT and department size, 2-tailed Wilcoxon Matched-Pair test for dependent variables and 2-tailed Mann–Whitney U-test for independent variables to calculate group differences. The level of significance was set at p < 0.05.

**Results:**

Return rates were: D: TE 89/215 (41.39%), TR 78/184 (42.39%); CH: TE 19/25 (76%), TR 22/30 (73.33%). Cronbach’s alpha values for TE scales were >0.8 or >0.9, and >0.7 to >0.5 for TR scales. Swiss hospitals surpassed German ones significantly in Global Satisfaction with AM (TR and TE); Clinical Competency training in CON (TE) and AM (TE, TR), Error Management, Culture of Decision Making, Evidence-based Medicine, and Clinical Competency in internal medicine CON and AM (TE). When the comparison was restricted to departments of comparable size, differences remained significant for Clinical Competencies in AM (TE, TR), and Culture of Decision Making (TE). CON received better grades than AM in Global Satisfaction and Clinical Competency. Quality of PGMT depended on department size, working conditions and structural training features.

**Conclusion:**

The lower quality of PGMT in German hospitals can be attributed to larger departments, more difficult working conditions, and less favorable structural features for PGMT in AM, possibly also in relation to increased financial pressure.

## Background

As the use and acceptance of complementary and alternative medicine (CAM) is growing amongst the public and health professionals [1,2], its status within the public healthcare system is also becoming more official. In 2002 58% of the Swiss population demanded more CAM for the future of medicine [3], and in 2009 67% accepted an amendment to the national constitution declaring CAM to be a matter of official policy [4]. This was tied to demands for an improved official status of CAM disciplines practiced by physicians, i.e. in research, medical education, health insurance, drug legislation and certification of health professionals [5]. There have been frequent studies of the reasons for the use of CAM. They include a need for additional or less toxic therapeutic options especially in chronic and incurable diseases, improved quality of life, a better doctor-patient relationship with more time for patients and more holistic care [6-8]. Indeed, a comparative Swiss national survey in 2006 showed that patients of medical practices providing CAM document longer-lasting and more severe health problems, but also higher overall patient satisfaction with treatments than patients of conventional (CON) practices [9]. This was especially true for practices offering anthroposophic medicine (AM) [10]. The public wish for more CAM includes stationary care: the majority would prefer CAM to CON hospitals given equal success rates [11] and opt for public financial support of CON hospitals offering CAM [12]. This shows that what is at stake is a meaningful integration of CAM into CON within the public healthcare system, including stationary forms of care.

If this is to happen, however, integrative medicine (IM) needs to become an integral element of undergraduate and postgraduate medical education, in addition to the need to create an appropriate evidence base for the safety and effectiveness of CAM methods as such. Also, in the spirit of evidence-based medical education, the evaluation of quality and effectiveness of IM education will be necessary. A majority of those responsible for representing medical schools in Germany, Switzerland and Austria as well as their medical students favor the integration of CAM into the medical system, but only a limited number of courses on CAM have been introduced and evaluated in undergraduate medical education [13-15]. A promising development is the creation and controlled evaluation of a clinical education ward for IM at Witten/Herdecke University, Germany, where final year undergraduate medical students learn to take active care of patients under supervision in an IM setting, integrating anthroposophic medicine (AM) into CON, with a positive impact on the quality of care [16].

In the field of postgraduate medical training (PGMT) an increasing need is articulated to include some forms of IM [17], but so far little has been done to evaluate PGMT in IM except for an internet-based on-line IM training program implemented in family residency programs in the United States [18]. In Germany and Switzerland some CON hospitals offer forms of CAM [19,20] and some of these take part in PGMT. Indeed, PGMT in an on-ward form can be expected to be the richest and most effective form of training in IM, because it usually provides more systematic, interdisciplinary and scientific learning formats in theory and practice, combined with responsible patient work for more challenging patients, under direct supervision of a more diversified array of experienced trainers in comparison to medical practices. Additionally, AM hospitals usually have a more complete set of specific pharmacological and non-pharmacological AM treatments than medical practices can offer [21].

AM hospitals in Germany and Switzerland have longstanding and substantial experience in PGMT in IM and can serve as models for such training. AM is an integrative form of CON which has developed since the 1920s [22]. It is based on a holistic concept that takes account of physical, living, emotional, cognitive, spiritual and social aspects in theory and practice [23]. It is practiced by conventionally trained physicians with an additional training in AM. In Germany and Switzerland AM hospitals are well integrated in the public healthcare systems and are accredited for the official PGMT of physicians in both countries [24-26]. PGMT in AM hospitals includes CON and AM aspects in a completely integrative way, i.e. conceptually as well as practically. However, so far an evaluation of IM training in AM hospitals is lacking.

For this reason and in view of public interest in IM we conducted a comprehensive cross-sectional evaluation of the subjectively perceived quality of CON and AM aspects of PGMT among trainees and trainers in all AM hospitals in Switzerland and Germany. In this paper we report on the results relating to the basic dimensions of PGMT, the working situation and the specific IM learning culture and teaching structure. The analyses are differentiated for Germany and Switzerland and for department sizes and clinical disciplines but not for single departments or hospitals. In addition, in order to provide a differentiated basis for possible improvements to PGMT in AM hospitals, we performed detailed quantitative and qualitative analyses of specific problems in IM training in PGMT as well as problem-solving options from the viewpoints of trainers and trainees. For reasons of space these results have to be published in two additional separate papers.

## Methods

### Survey and institutions

We conducted an anonymous questionnaire-based cross-sectional complete survey, i.e. among all trainer and trainee physicians in all eleven German and four Swiss anthroposophic hospitals. The survey was carried out from July - December 2010, and the first data analyses performed in spring 2011. However, due to a lack of resources and especially of personnel (pregnancy and motherhood in one case and change of institutions in another), data processing and the preparation of the manuscript were delayed, and submission was only possible at the end of 2013. Nevertheless our results still describe the first and only evaluation of its kind and have remained highly valid for PGMT in AM. Our survey included 215 trainees and 184 trainers in Germany and 25 trainees and 30 trainers in Switzerland. In Switzerland the survey took place within the framework of the annual national PGMT quality assessment of the Swiss Federation of Physicians (FMH), carried out by the Swiss Federal Institute of Technology Zurich (Eidgenössische Technische Hochschule Zürich, ETHZ). In Germany it was undertaken in agreement with the German Medical Association (Bundesärztekammer) in Berlin, but for logistical reasons outside regular national assessments, and always carried out by ETHZ. The paper and pencil questionnaires were distributed personally to each trainer and trainee who, upon completion, sent them directly back in a pre-stamped envelope, in Switzerland to the ETHZ, in Germany to WHU, which collected them and sent them on to the ETHZ. The survey was anonymous for the physicians and semi-anonymous for the institutions, with codes for the training departments and clinical disciplines but without identification of respondents. All quantitative data analyses were carried out at ETHZ, and all free-text answers were analyzed qualitatively at WHU. All data were handled according to the German and Swiss data protection laws. In Germany, the adherence of the procedure to data protection law was cross-checked by the data-protection officer of WHU. As in all the other national and regular surveys on the quality of PGMT in Swiss and German hospitals carried out by the ETHZ over the past 10 years in cooperation with the Swiss Federation of Physicians and the German Medical Association and in accordance with German and Swiss national standards, there was no need to obtain an ethics approval from an ethics committee and informed written consent from the participants prior to the assessment for this study, because a survey of this kind for educational quality assessment - which is not accompanied by physical or psychological burden - is explicitly excluded from the definition of “research projects on human subjects” for which an ethics approval is necessary [27].

### Questionnaire

The assessment instrument was a paper and pencil questionnaire in two versions, one for trainees and one for trainers, each consisting of two parts. The first part was identical to the questionnaire that had been developed and validated in Zurich for the annual PGMT quality assessment in hospitals in Switzerland and Germany. The questionnaire was first developed in 2003 for the annual national assessment of the subjectively perceived quality of postgraduate medical education in all hospitals in Switzerland. Content validity was ascertained in cooperation with the national steering board for postgraduate medical education, consisting of the officers in charge and trainees of the different medical disciplines. After the annual data assessments, item analyses were performed and the optimized questionnaire submitted to and approved by the steering board. The questionnaire contained eight pages with 71 questions for trainees, and eight pages with 67 questions for trainers [28]. The major subject domains of this part in both versions covered questions on general (CON) aspects of PGMT, including Global Satisfaction with residency training (4 questions), Clinical Competencies (28), Learning Culture (7), Leadership Culture (6), Error Management (4), Culture of Decision Making (4), Department Culture (4), and Evidence-Based Medicine (4). Further questions (12) included the working situation and baseline characteristics of respondents as well as a module on respondents’ attitudes towards CAM [29]. The main answer format for the questions consisted of a 6-point scale corresponding to the national school grade systems, i.e. 1 for best and 6 for worst scores in Germany, and the inverse in Switzerland. Swiss scales were recoded to the German system for calculation purposes.

The second part of the questionnaire was developed by the WHU authors (SE, PH) jointly with ETHZ (PO) to assess AM aspects of PGMT and their integration in CON PGMT [30]. It consisted of four pages with 22 questions for the trainees and three pages with 20 questions for the trainers. The major domains in this part in both versions covered Overall Satisfaction (4 questions for trainees; 3 for trainers), Clinical Competencies in AM (2, with 16 sub-questions for trainees; 1, with 9 sub-questions for trainers), integration of AM and CON (2; 2), teaching and continuing education (5; 7), structural problems and problem solving (including free-text answers) (5; 4), and personal data (2; 2). The questions about the working situation, integration and structural problems were included as important indicators for educational quality because the features they covered were considered to be prerequisites for optimal integrative medical education: a working situation that allows for the completion of work as well as continuing education during instead of outside contractually agreed working hours, an active integration of conventional and complementary elements through functioning role models provided by trainers as well as in daily practice, and department structures that include regular events explicitly related to integrative medicine such as personal training career supervision, bedside teaching, educational events or study groups as well as the time to take part in these events.

### Data analysis

Quantitative data were analyzed descriptively at ETHZ, allowing for an a priori defined comparison between a) Swiss and German AM hospitals, b) CON and AM aspects of PGMT, c) trainers and trainees, d) clinical disciplines, and e) department sizes. As our survey was not carried out on random samples or subsets of the targeted populations but consisted of a census, covering the whole cross-sectional population of trainees and trainers in PGMT in AM hospitals in Germany and Switzerland, interferential statistics such as multivariate analyses were not planned. Also, due to the very small size of the relevant samples (often considerably fewer than ten), multivariate analyses were not possible. Pearson correlation was calculated (two-tailed) to investigate the relationship between specific quality dimensions of PGMT and department size as defined by numbers of trainees per department. Inter-group differences were calculated using the two-sided Wilcoxon matched-pair test for dependent variables and the Mann–Whitney U-test for independent variables (the different distributions deviated from normality). The level of significance was set at p < 0.05. In order to calculate the statistical difference between Global Satisfaction scores of trainers and trainees, the scale for trainees had to be adapted (reduction from four to three items) in order to be identical with the scale for trainers (three items). A statistical comparison of Clinical Competency in AM and CON was not possible because of content differences between the different scales, and a statistical comparison of the trainee and trainer scores for Clinical Competency was not possible due to the different direction of the respective questions. To calculate the overall score differences in basic PGMT dimensions between Germany and Switzerland, we included a comparison of departments of comparable size in both countries (seven or less trainees per department, corresponding to seven as being the highest number of trainees in the largest Swiss department), because PGMT dimensions correlated with department size and, in contrast to Germany, Swiss hospitals had only small and medium-sized but no large departments (predefined as 1–3, 4–10 and 11 or more trainees per department). Cronbach’s alpha was calculated for internal consistency of the questionnaire scales.

## Results and discussion

### Return rate

In Germany, 89/215 (41.39%) questionnaires for trainees and 78/184 (42.39%) for trainers were returned, in Switzerland the corresponding numbers were 19/25 (76%) for trainees and 22/30 (73.33%) for trainers.

### Baseline characteristics of respondents

The baseline characteristics of both trainers and trainees differed considerably in the two countries. Whereas senior physicians constituted the majority of trainers in Germany, the majority in Switzerland were assistant medical directors and medical directors (Table 1).

**Table 1:** Baseline characteristics of trainers in Germany and Switzerland

**Trainers**	**Germany**		**Switzerland**	
**Function**	**N**	**Valid%**	**N**	**Valid%**
Senior physician	45	60.0	5	22.7
Staff specialist	4	5.3	2	9.1
Assistant medical director	15	20.0	10	45.5
Medical director	9	12.0	5	22.7
Other	2	2.7	0	0

Amongst the trainees the mean year of graduation differed by only about three months, but in Germany the completed mean duration of postgraduate medical education was one year longer than in Switzerland and the mean duration of work in their departments 18 months longer (Table 2). This was due to a small number of relatively older physicians in a resident position for over ten years (N = 13, 14.4%), whereas in Switzerland no one exceeded nine years (details not shown). Age was not recorded in order to avoid possible identification and thus violate the anonymity of respondents. In addition, trainees in Switzerland had a more international background (the majority actually coming from Germany), a higher percentage of females and of full-time employees, and most (71%) intended to specialize in general or family medicine, whereas their colleagues in Germany had broader aims for different specialties, the largest portion (24%) for internal medicine.

**Table 2:** Baseline characteristics of trainees in Germany and Switzerland

	**Germany**		**Switzerland**	
**Educational status**	N	Mean (SD)	N	Mean (SD)
Year, Graduation from university	84	2003.21 (6.65)	19	2002.95 (6.81)
Years, duration of postgraduate medical education	88	4.93 (5.02)	18	3.96 (2.95)
Months, duration of working in this department	85	25.76 (26.21)	17	7.94 (4.16)
**Gender**	Male	Female	Male	Female
	33 (38.4)	53 (61.6)	5 (26.3)	14 (73.7)
**Intended specialty**	N (valid%)		N (valid%)	
Family/general internal medicine	11 (14.7)		10 (71.4)	
Internal medicine (incl. subspecialties)	18 (24.0)		2 (14.3)	
Gynecology & obstetrics	12 (16.0)		2 (14.3)	
Pediatrics	8 (10.7)		-	
Psychiatry, psychosomatics, psychotherapy.	14 (15.6)		-	
others*	12 (15.8)		-	
**Employment**				
Full-time (95% or more)	13 (16.0)		14 (73.7)	
Part-time (76-94%)	63 (77.8)		2 (10.5)	
Part-time (50-75%)	5 (6.2)		3 (15.8)	
**Country of graduation**				
Germany	81 (94.2)		10 (52.6)	
Switzerland	1 (1.2)		4 (21.1)	
other	4 (4.7)**		5 (26.3)***	

*Anesthesiology; general, orthopedic & traumatic surgery, neurosurgery; neurology, pediatric & adolescent psychiatry, radiology: **Italy; other. ***Austria & other European Union; other.

### Reliability of questionnaire scales

In both countries, in all trainee answers Cronbach’s alpha for the scales of the major dimensions were excellent (>0.90) or good (>0.80) (except for Error Management in Germany) (Table 3); they were also excellent in trainers’ ratings of Global Satisfaction and Clinical Competencies in AM. However, except for Global Satisfaction with CON and Evidence-based Medicine in Switzerland, alpha-values of the trainers were not more than acceptable (>0.70), in some cases even questionable (>0.60) or low (>0.50).

**Table 3:** Cronbach’s alpha of the questionnaire scales for the basic dimensions of postgraduate medical training

**Quality dimensions**	**Trainees in Germany**	**Trainees in Switzerland**	**Trainers in Germany**	**Trainers in Switzerland**
**Global Satisfaction CON**	0.94	0.95	0.69	0.88
**Global Satisfaction AM**	0.92	0.89	0.92	0.94
**Clinical Competencies CON**	0.95	0.96	0.75	0.74
**Clinical Competencies AM**	0.93	0.89	0.94	0.96
**Learning Culture**	0.91	0.93	0.53	0.76
**Leadership Culture**	0.92	0.93	0.61	0.73
**Error Management**	0.6	0.87	0.57	0.79
**Culture of Decision Making**	0.87	0.86	0.72	0.67
**Department Culture**	0.88	0.95	0.6	0.78
**Evidence-based Medicine**	0.91	0.95	0.76	0.89

### Global satisfaction and clinical competency training in conventional and anthroposophic postgraduate medical training in Germany and Switzerland, perceived by trainees and trainers

**Figure 1:**
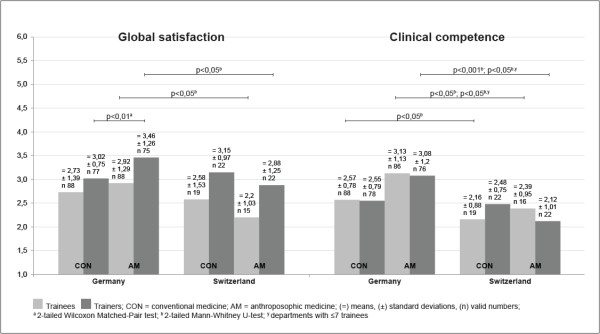
**Global satisfaction with and quality judgment of competency training in conventional (CON) and anthroposophic (AM) aspects of postgraduate medical training in Germany and Switzerland.** Rating by trainees (light bars) and trainers (dark). 1 indicates the highest, 6 the lowest possible degree of perceived satisfaction or quality. Means, standard deviations and valid numbers of respondents; statistically significant differences between indicated groups. (for U-values consult text).

In Germany, the trainers expressed significantly more mean Global Satisfaction with CON aspects of PGMT compared to AM aspects (two-sided Wilcoxon Matched-Pair Test, U = -2.87, p < 0.01 [Figure 1, bars on left side]). Other graphically visible differences were not significant: in both countries more mean Global Satisfaction of trainees compared to trainers in CON and AM, and in Switzerland better scores for AM than CON as rated by trainees as well as trainers. In the comparison of both countries, the trainees’ as well as the trainers’ Global Satisfaction with AM aspects of PGMT was significantly lower in German than in Swiss hospitals (p < 0.05, 2-tailed Mann–Whitney U-test, trainees: U = -2.03, trainers: U = - 1.97) . This significance was lost when only the departments of comparable size (seven or less trainees) were compared (details not shown).A statistical score comparison of CON with AM competencies and of trainee and trainer scores was not possible for Clinical Competency training (see Data analysis section). In Germany (but not Switzerland) the mean training quality for Clinical Competencies received a similar rating from trainees and trainers, and it was better for CON than AM competencies (Figure 1, bars on right side). However, the statistical comparison of like scales and groups in both countries revealed that in Switzerland the Clinical Competency training for CON received significantly better grades from trainees than in Germany (2-tailed Mann–Whitney U-test, U = -2.34, p < .05) and that the same was true for AM as perceived by trainees (U = -2.46, p < .05) as well as by trainers (U = -3.68, p < .001). When calculated for departments of comparable size only, the country differences for Clinical Competencies in AM remained significant both for trainees (U = -1.97, p < .05; Germany, mean ± SD: 2.99 ± 1.08, n = 45; Switzerland: 2.39 ± 0.95, n = 16) as well as trainers (U = -3.56, p < .001; Germany: 3.21 ± 1.29, n = 52; Switzerland: 2.12 ± 1.01, n = 22).

### Basic dimensions of postgraduate medical training and size of hospital departments in Germany and Switzerland

As the size of clinical departments as measured by the number of trainees per department varied considerably (range 1–19), and as many departments were only small (1–3 trainees) or medium-sized (4-10 trainees), a statistical comparison of department groups according to the predefined size groups “small”, “medium sized” or ”large” (11 and more) was not feasible. We examined the relationship of department size and the basic quality dimensions of PGMT as perceived by the trainees by calculating the Pearson-correlation (two-tailed testing) between them (Table 4). In German hospitals, there was a consistent positive correlation between increasing department size and decreasing quality. This was statistically significant for all dimensions except Clinical Competencies in AM, Error Management, and Evidence-based Medicine. In Switzerland however, department size correlated with increasing quality, albeit without statistical significance. Unlike Germany, Swiss hospitals had no large, but only medium-sized and small clinical departments. Compared to Germany, trainees in Swiss hospitals not only gave significantly better scores for Global Satisfaction with AM as well as Clinical Competency training in CON and AM (see above, and Table 4), but also for Error Management, Culture of Decision Making and Evidence-based Medicine. However, if calculated for departments of comparable size, apart from Clinical Competency training in AM (see above), only the difference for Culture of Decision Making remained significant (Table 4).

**Table 4:** Basic dimensions of postgraduate medical training as perceived by trainees and size of hospital departments in Germany and Switzerland

**Basic dimensions of postgraduate medical training**	**German hospitals**		**Swiss hospitals**		**Comparison of German and Swiss departments**
	**Mean ± SD, (valid n); *Values for dpts. ≤ 7***	**Correlation coefficient**^**a**^**, p-value**	**Mean ± SD, (valid n) *Values for dpts. ≤ 7***	**Correlation coefficient**^**a **^**p-value**	**U-value**^**b**^**, p-value *Values for dpts. ≤ 7***
**Global Satisfaction CON**	2.73 ± 1.39, (88)	r = .38, p < 0.001	2.58 ± 1.53, (19)	r = -.12, n.s	U = -.63, n.s.
	*2.41 ± 1.27, (46)*		*2.58 ± 1.53, (19)*		*U = -.19, n.s.*
**Global Satisfaction AM**	2.92 ± 1.29, (88)	r = .32, p < 0.01	2.2 ± 1.03, (15)	r = -.26, n.s.	U = -2.03, p < .05
	*2.63 ± 1.11, (46)*		*2.2 ± 1.03, (15)*		*U = -1.29, n.s.*
**Clinical Competencies CON**	2.57 ± 0.78, (88)	r = .31, p < 0.01	2.16 ± 0.88, (19)	r = -.22, n.s	U = -2.34, p < .05
	*2.47 ± 0.78, (46)*		*2.16 ± 0.88, (19)*		*U = -1.68, n.s*
**Clinical Competencies AM**	3.13 ± 1.13, (86)	r = .13, n.s.	2.39 ± 0.95, (16)	r = -.36, n.s.	U = -2.46, p < .05
	*2.99 ± 1.08, (45)*		*2.39 ± 0.95, (16)*		*U = -1.97, p < .05*
**Learning Culture**	2.54 ± 1.05, (88)	r = .34, p < 0.01	2.32 ± 1.2, (19)	r = -.07, n.s.	U = -1.06, n.s.
					*U = -.34, n.s.*
	*2.29 ± 0.91, (45)*		*2.32 ± 1.2, (19)*		
**Leadership Culture**	2.6 ± 1.11, (88)	r = .39, p < 0.001	2.49 ± 1.22, (19)	r = -.11, n.s.	U = -.55, n.s.
	*2.38 ± 1.03, (46)*				*U = -.24, n.s*.
			*2.49 ± 1.22, (19)*		
**Error Management**	2.83 ± 1.22, (89)	r = .09, n.s.	2.32 ± 1.43, (19)	r = -.45, n.s.	*U = -1.97, p < .05*
	*2.80 ± 1.26, (46)*		*2.32 ± 1.43, (19)*		*U = -1.79, n.s.*
**Culture of Decision Making**	2.13 ± 1.01, (88)	r = .36, p < 0.01	1.53 ± 0.9, (19)	r = -.21, n.s.	U = -3.03, p < .01
	*1.93 ± 0.81, (45)*		*1.53 ± 0.9, (19)*		*U = -2.44, p < .05*
**Department Culture**	2.12 ± 0.98, (88)	r = .53, p < 0.001	2.28 ± 1.53, (19)	r = -.27, n.s.	U = -.48, n.s.
	*1.78 ± 0.74, (46)*		*2.28 ± 1.53, (19)*		*U = -.58, n.s*.
**Evidence-based Medicine**	4.41 ± 1.19, (88)	r = .17, n.s.	*3.57 ± 1.76, (19)*	r = -.44, n.s.	U = -2.00, p < .05
	*4.39 ± 1.21, (45)*				*U = -1.83, n.s.*

^a^Pearson-Correlation, 2-tailed; ^b^2-tailed Mann–Whitney U-test; dpts. = departments; SD = standard deviation; r = correlation coefficient; n.s. = not significant; CON = conventional medicine; AM = anthroposophic medicine.

Means and standard deviations of rating scores: 1 indicates the highest, 6 the lowest possible degree of quality; numbers of trainees; Pearson-correlation between the basic quality dimensions of PGMT and size of hospital departments as measured by the numbers of trainees per department, a positive r-value signifying a linear correlation between decreasing quality and increasing department size; all-over comparison of German and Swiss departments as well a comparison of similar-sized departments (≤7 trainees per department, in italics).

### Global satisfaction, quality of clinical competency training and clinical disciplines in German and Swiss hospitals, perceived by trainees

In most clinical disciplines of German anthroposophic hospitals, mean Global Satisfaction with CON aspects of PGMT was higher than with AM aspects: AM only received better mean scores than CON in internal medicine and the same was true for internal medicine in Swiss hospitals (Table 5). Yet these differences were statistically significant only for gynecology and obstetrics. As regards the quality of Clinical Competency training, practically all disciplines in both countries – except gynecology and obstetrics in Switzerland – had better mean scores for CON than for AM aspects of PGMT. Due to different questions in the Clinical Competency scales for CON and AM, no statistical comparisons could be made between these features. The comparison of the quality of Clinical Competency training in internal medicine in German and Swiss hospitals showed significantly better scores for Switzerland both in CON and AM aspects of internal medicine.

**Table 5:** Global satisfaction and quality of clinical competency training as perceived by trainees, and clinical disciplines in German and Swiss hospitals

**Clinical disciplines**	**CON in German hospitals**	**AM in German hospitals**	**CON in Swiss hospitals**	**AM in Swiss hospitals**	**U-value**^**a**^**, p-value**^**b**^	**U-value**^**a**^**, p-value**^**c**^	**U-value**^**d**^**, p-value**^**e**^	**U-value**^**d**^**, p-value**^**f**^
*Global Satisfaction*^*g*^								
Anesthesiology	2,13 ± 0,72 (4)	3,08 ± 1,01 (3)	n.a.	n.a.	n.a.	n.a.	n.a.	n.a.
Surgery	2,85 ± 1,78 (5)	3,35 ± 1,75 (5)	n.a.	n.a.	n.a.	n.a.	n.a.	n.a.
Gynecology & Obstetrics	1,77 ± 0,52 (13)	2,48 ± 1,05 (13)	3,17 ± 2,47 (3)	1,63 ± 0,18 (2)	-1,97; <0,05	n.a.	n.a.	n.a.
Internal Medicine	3,23 ± 1,47 (33)	2,94 ± 1,39 (33)	2,25 ± 1,47 (12)	2,0 ± 0,99 (10)	-1,85; n.s.	-1,37; n.s.	-2,10; <0,05	-1,91; n.s.
Pediatrics	3,41 ± 1,06 (11)	3,57 ± 1,18 (11)	n.a.	n.a.	-0,72; n.s.	n.a.	n.a.	n.a.
Psychiatry & Psychotherapy	2,60 ± 1,52 (15)	2,64 ± 1,37 (16)	n.a.	n.a.	-0,25; n.s.	n.a.	n.a.	n.a.
Other disciplines	1,61 ± 0,5 (7)	2,89 ± 0,83 (7)	3,13 ± 0,97 (4)	3,25 ± 0,9 (3)	n.a.	n.a.	n.a.	n.a.
**All disciplines**	**2,73 ± 1,39 (88)**	**2,92 ± 1,29 (88)**	**2,58 ± 1,53 (19)**	**2,2 ± 1,03 (15)**	**n.a.**	**n.a.**	**n.a.**	**n.a.**
*Clinical Competencies*^*g*^								
Anesthesiology	2,31 ± 0,65 (4)	4,0 ± 0,94 (3)	n.a.	n.a.	n.a.	n.a.	n.a.	n.a.
Surgery	2,81 ± 0,77 (5)	3,73 ± 1,81 (5)	n.a.	n.a.	n.a.	n.a.	n.a.	n.a.
Gynecology & Obstetrics	2,14 ± 0,44 (13)	3,09 ± 0,98 (13)	2,59 ± 0,94 (3)	1,72 ± 0,39 (2)	n.a.	n.a.	n.a.	n.a.
Internal Medicine	2,81 ± 0,95 (33)	3,05 ± 1,08 (33)	1,91 ± 0,78 (12)	2,14 ± 0,78 (10)	n.a.	n.a.	-2,95; <0,01	-2,46; <0,05
Pediatrics	2,72 ± 0,5 (11)	3,47 ± 0,78 (11)	n.a.	n.a.	n.a.	n.a.	n.a.	n.a.
Psychiatry & Psychotherapy	2,54 ± 0,78 (15)	2,63 ± 1,27 (16)	n.a.	n.a.	n.a.	n.a.	n.a.	n.a.
Other disciplines	2,07 ± 0,21 (7)	3,44 ± 1,12 (5)	2,58 ± 1,07 (4)	3,35 ± 0,94 (4)	n.a.	n.a.	n.a.	n.a.
**All disciplines**	**2,57 ± 0,78 (88)**	**3,13 ± 1,13 (86)**	**2,16 ± 0,88 (19)**	**2,39 ± 0,95 (16)**	**n.a.**	**n.a.**	**n.a.**	**n.a.**

^a^2-tailed Wilcoxon Matched-Pair test for dependent variables.

^b^Comparison of CON and AM in German hospitals.

^c^Comparison of CON and AM in Swiss hospitals.

^d^2-tailed Mann–Whitney U-test for independent variables.

^e^Comparison of CON in German hospitals and CON in Swiss hospitals.

^f^Comparison of AM in German hospitals and AM in Swiss hospitals.

^g^Mean ± standard deviation (valid numbers of trainees), n.a. = not applicable; n.s. = not significant; CON = conventional medicine; AM = anthroposophic medicine.

Other disciplines = Family Medicine in Switzerland; Neurology, Neurosurgery and Radiology in Germany.

1 indicates the highest, 6 the lowest possible degree of perceived satisfaction or quality.

### Working situation in German and Swiss anthroposophic hospitals

As an indicator of the perceived working situation in the departments studied we used the self-indicated ability of the trainees to complete their work as well as their continuing education during the contractually agreed working hours. Table 6 shows better mean scores for completion of work as well as continuing education in Switzerland. However, when scores were correlated to department size, German departments showed a less favorable working situation for larger departments which is statistically significant for work completion and displayed a trend (p < 0.1) for continuing education. If only departments of a comparable size were compared for both countries (seven or less, corresponding to the largest Swiss department size of seven trainees), then Switzerland scored significantly better for completion of continuing education.

**Table 6:** Working situation in German and Swiss anthroposophic hospitals as indicated by the trainees’ self-reported ability to complete their work as well as their continuing education during contractually agreed working hours

**“During the contractually agreed working hours I can complete my work to my full satisfaction”** (1 indicates: “fully applies”, and 6: “does not apply at all”)		
	**German hospitals**	**Swiss hospitals**
Mean ± standard deviation, (n) = valid numbers of trainees of all departments	3.75 ± 1.61 (89)	2.90 ± 1.60 (19)
Correlation^a^ of score with department size, p-value	r = 0.29, p < 0.01	r = 0.38, n.s.
Mean ± standard deviation, (n) = number of trainees of departments with 7 or less trainees	3.50 ± 1.57 (46)	2.90 ± 1.60 (19)
	**Comparison of German and Swiss departments of comparable size**	
Comparison of departments with 7 or less trainees. U-value^b^, p-value	U = -1.41, n.s.	
**“During the contractually agreed working hours I can complete my continuing education to my full satisfaction”** (1 indicates: “fully applies”, and 6: “does not apply at all”)		
	**German hospitals**	**Swiss hospitals**
Mean ± standard deviation, (n) = valid numbers of trainees of all departments	4.3 ± 1.43 (89)	3.37 ± 1.67 (19)
Correlation^a^ of score with department size, p-value	r = 0.19, n.s. (p < 0.1)	r = 0.15, n.s.
Mean ± standard deviation, (n) = number of trainees of departments with 7 or less trainees	4.22 ± 1.25, (46)	3.37 ± 1.67 (19)
	**Comparison of German and Swiss departments of comparable size**	
Comparison of departments with 7 or less trainees. U-value, p-value^b^	U = -2.02, p < 0.05	

^a^Pearson Correlation, 2-tailed; r = correlation coefficient; n.s. = not significant; ^b^2-tailed Mann–Whitney U-test.

Correlation between rating scores and department size (number of trainees per department) and comparison between German and Swiss departments of comparable size. A lower score corresponds to a better working situation.

### Integrative learning culture in German and Swiss anthroposophic hospitals

As indicators for an integrative learning culture that fosters trainees’ competencies to integrate AM elements into CON in clinical practice, we used the questions shown in Table 7, enquiring about trainers as integrative role models and about the combination of CON and AM in daily clinical work. In Switzerland, department size correlated moderately with both aspects of integrative learning culture, suggesting some quality increase with increasing department size, albeit without statistical significance. In Germany there was practically no correlation between department size and integrative learning culture. However, although Swiss departments showed a better mean rating of perceived integrative role models and of integrative clinical practice, the difference between departments of comparable size in both countries was not significant.

**Table 7:** **Integrative learning culture in German and Swiss anthroposophic hospitals** as indicated by perceived role models for the integration of conventional and anthroposophic medicine in daily clinical practice and by inclusion of AM in daily work

**“In my department the trainers are a good role model for complementing conventional medicine with anthroposophic medicine in daily clinical work”.** 1 indicates: “fully applies”, and 6: “does not apply at all”.		
	**German hospitals**	**Swiss hospitals**
Mean ± standard deviation, (n) = valid numbers of trainees of all departments	2,60 ± 1,38 (87)	1,47 ± 0,92 (15)
Correlation^a^ of score with department size, p-value	r = 0.06, n.s.	r = -0.30, n.s.
Mean ± standard deviation, (n) = number of trainees of departments with 7 or less trainees	2.42 ± 1.37 (45)	1.47 ± 0.92 (15)
	**Comparison of German and Swiss departments of comparable size**	
Comparison of departments with 7 or less trainees. U-value^b^, p-value	U = -0.83, n.s.	
**“In my department conventional medicine and anthroposophic medicine are equally included in daily clinical work”.** 1 indicates: “fully applies”, and 6: “does not apply at all”.		
	**German hospitals**	**Swiss hospitals**
Mean ± standard deviation, (n) = valid numbers of trainees of all departments	2,62 ± 1,48 (87)	1,44 ± 0,63 (16)
Correlation^a^ of score with department size, p-value	r = 0.12, n.s.	r = -0.36, n.s.
Mean ± standard deviation, (n) = number of trainees of departments with 7 or less trainees	2.58 ± 1.57 (45)	1.44 ±, 0.63 (19)
	**Comparison of German and Swiss departments of comparable size**	
Comparison of departments with 7 or less trainees. U-value^b^, p-value	U = -0.73, n.s.	

^a^Pearson Correlation, 2-tailed; r = correlation coefficient; n.s. = not significant; ^b^2-tailed Mann–Whitney U-test.

Trainees’ ratings (a lower score corresponds to a better integration); correlation between rating score and department size (number of trainees per department); comparison between German and Swiss departments of comparable size.

### Integrative training structures in German and Swiss anthroposophic hospitals

In order to assess structural department features that foster the integrative aspects of PGMT in AM hospitals, we asked trainees about regular personal career supervision in AM, educational events, bedside teaching, study or working groups in AM, and the time to take part in them (Table 8). The results indicate that Swiss AM hospitals provide a higher rate and a higher frequency of regular personal career supervision in AM, more or longer regular educational events during working hours, more regular bedside teaching in AM and more AM study or working groups, and that Swiss trainees have more time to take part in them.

**Table 8:** Structural department features for the training of anthroposophic medicine within postgraduate medical education in Germany and Switzerland as perceived by trainees

**“Does your postgraduate medical training in anthroposophic medicine include regular personal training career supervision?”**		
If yes: every 6 months, every 12 months?		
	**German hospitals**	**Swiss hospitals**
**Yes**	12 (14.6%) (every 6 months)	9 (56.3%) (every 6 months)
	20 (24.4%) (every 12 months)	3 (18.8%) (every 12 months)
**No**	50 (61.0%)	4 (25%)
**“Does your postgraduate medical training department offer regular educational events during working hours (courses, seminars, lectures)?”**		
If yes: hours per week, mean ± SD (n)		
	**German hospitals**	**Swiss hospitals**
**Yes**	62 (72.9%)	10 (90.9%)
	Hours: 1.73 ± 1.56 (59)	Hours: 2.52 ± 1.43 (15)
**No**	23 (27.1%)	1 (9.1%)
**“Does your postgraduate medical training department organize regular bedside teaching in anthroposophic medicine?”**		
If yes: hours per week, mean ± SD (n)		
	**German hospitals**	**Swiss hospitals**
**Yes**	25 (29.4%)	9 (60%)
	Hours: 2.63 ± 2.04 (24)	Hours: 2.50 ± 1.73 (9)
**No**	50 (58.8%)	6 (40%)
**Don’t know**	10 (11.8%)	0 (0%)
**“Does your postgraduate medical training department offer anthroposophic medical study or working groups?”**		
Hours per week, (%)		
	**German hospitals**	**Swiss hospitals**
**Yes**	60 (74.1%)	15 (100%)
**No**	12 (14.8%)	0 (0%)
**Don’t know**	9 (11.1%)	0 (0%)
**“If yes, do you have the time to take part in these study or working groups?”**		
Answer options aggregated: Yes = “yes” + “rather yes”; No = “rather no” + “no”		
Hours per week, (%)		
	**German hospitals**	**Swiss hospitals**
**Yes**	30 (43.5%)	11 (73.3%)
**No**	39 (56.5%)	4 (26.7%)

The table shows valid numbers and percentage of responding trainees (in brackets); means ± standard deviations (SD) of hours.

This is the first study worldwide to evaluate the quality of PGMT in hospitals that integrate CON and CAM within public healthcare systems, to the best of our knowledge. We chose AM as a form of CAM with a European origin because of its well-known integrative care culture in theory, practice and medical training as well as for its longstanding experience in PGMT within the public healthcare systems in Germany and Switzerland. Apart from this, the strengths of this study lie in a) the focus on one form of IM only, allowing for a defined profile of IM, namely AM; b) its full survey of all the trainees and trainers in PGMT in all AM hospitals in Germany and Switzerland, the countries with the largest numbers of AM hospitals worldwide; c) the broad assessment of PGMT quality, including the official instrument for the national yearly PGMT assessments in all hospitals of both countries as well as a specific instrument for AM; and c) its direct link to two consecutive investigations on specific problems and problem solving options for integrative PGMT in AM hospitals.

However, our study also has limits. a) A weakness of the study is the low return rate from trainees (41%) and trainers (42%) in Germany; and in Switzerland the advantage of higher return rates (trainees 76%, trainers 73%) is hampered by the small absolute numbers of respondents, so that selection and detection bias cannot be excluded. b) The absolute numbers in the different groups (disciplines and country comparisons) differed considerably, and the baseline characteristics of the respondents varied greatly with respect to the professional status of the trainers as well as gender, part-time work, national background, duration of resident status and intended specialty (Tables 1 and 2). c) Also, due to small sample sizes, multivariate analyses were not possible (see Data analyses section). d) Due to a lack of personnel and resources, processing the data from 2010 was only possible after a delay. However, as this is still the first and only investigation of its kind and the situation of PGMT in AM hospitals has basically remained the same or deteriorated due to increasing economic pressure (see Discussion), our results are still highly valid for PGMT in AM hospitals.

With regard to Global Satisfaction with and perceived quality of Clinical Competency training, Swiss anthroposophic hospitals surpassed German ones due to significantly higher Global Satisfaction with and significantly better Clinical Competency training in AM aspects of PGMT in the perception of trainers as well as trainees and, similarly, a significantly better Clinical Competency training in CON as viewed by trainees (Figure 1). At the level of clinical disciplines, internal medicine received significantly better ratings from trainees for Global Satisfaction with CON as well as for Clinical Competency training in CON and AM in Swiss than in German hospitals (Table 5). When the comparison was confined to departments of comparable size (seven or less trainees), statistical significance was retained for Clinical Competency in AM, but lost for the differences in Global Satisfaction with AM and in Clinical Competency training in CON (Table 4). The same phenomenon applies to Error Management, Culture of Decision Making and Evidence-based Medicine which scored significantly better in Swiss hospitals, but – with the exception of Culture of Decision Making – lost significance when only equal-sized departments were compared (Table 4).

The latter finding appears to be connected to the inverse correlation we found between department size and quality of basic PGMT dimensions. In German but not Swiss hospitals, increasing department size correlated consistently, i.e. in all ten quality dimensions, and significantly in seven of these, with decreasing quality of PGMT (Table 4). A plausible explanation for this correlation can be seen in the department size as such, with small sizes favoring close contacts and easier organization. This might save time and thus influence the workload. In contrast to Swiss anthroposophic hospitals, German hospitals not only had small and medium-sized (1–3 and 4–10 trainees, respectively) but also large-sized departments (11 or more trainees). In fact, our analysis of the working situation revealed a statistically significant negative correlation of department size, with reduced ability for work completion during official working time, and a similar trend for the completion of continuing education in Germany alone (Table 6). This concurs with our additional analysis of the problem with PGMT in AM hospitals which revealed insufficiently well-organized work as the next most important obstacle to optimal integrative PGMT after too heavy a work load and too much administrative work, especially in Germany [31].

The differences described between Germany and Switzerland may also be due in part to differences in healthcare systems. The DRG system, for example, existed in Germany at the time of our survey but not in Switzerland. In Germany, the DRG system has led to considerable dissatisfaction, forcing hospitals to extremely tight personnel schedules, a forced increase in case numbers, a drastically heavier work load and a critical shortage of funding [32,33]. It is highly likely that this situation has a significant impact on PGMT. This corresponds to the better working situation in Switzerland shown by our study, even for departments of comparable size (Table 6), and also with the results of our additional problem analysis: in Germany to a greater degree than in Switzerland, trainees as well as trainers identified excessive working hours, an overload of work and too much administrative work as the most important obstacles to optimal integrative PGMT with AM [31].

However, there must be also other factors than size and workload which are responsible for the reported differences between the two countries. In Switzerland, with only small and medium-sized departments, there was a statistically non-significant but consistently negative correlation between department size and scores for PGMT quality dimensions (a lower score indicating higher quality) (Table 4). In addition, the significantly better scores in Switzerland for Clinical Competencies in AM remained significant for both trainees and trainers, even when only departments of similar size are compared (Figure 1). A reason for this difference can be identified in the better structural department features aimed specifically at a systematic training of AM in PGMT: Swiss hospitals provided a higher rate and a higher frequency of regular personal career supervision related to AM, more or longer regular educational events during working hours, more regular bedside teaching in AM and more AM study or working groups, and trainees in Swiss hospitals have more time to take part in these (Table 8). Learning Culture, however, was not statistically different between the two countries, despite better mean ratings of perceived integrative role models and of the actual integration of AM into daily clinical practice amongst Swiss trainees (Table 7). Theoretically, Swiss trainers might be expected to have a higher level of knowledge and experience in AM, with relatively more assistant medical directors or directors in trainer functions compared to the senior physicians occupying this role in Germany (Table 1).

A last finding to discuss is the better mean ratings for Global Satisfaction and Clinical Competencies in CON compared to AM in most clinical disciplines, especially in Germany (Figure 1 and Table 5). The reasons for this are unclear. Quantitatively, CON elements play a much more important role in PGMT than AM, the elements of which provide but a complementary extension thereof [23]. Also, as already mentioned, suboptimal teaching structures and working conditions as well as their causes may lead to insufficient PGMT in AM and thus to reduced levels of Global Satisfaction and Clinical Competencies, a situation that we have identified as a plausible reason for the generally better quality of PGMT in Switzerland.

## Conclusion

In this first evaluation of on-ward PGMT in IM we examined the quality of PGMT as experienced by trainees and trainers in all anthroposophic hospitals in Germany and Switzerland (full survey). These hospitals are part of the national public healthcare systems. They use and teach the integration of AM into CON in this context. Swiss hospitals significantly exceeded German ones in basic dimensions of PGMT such as Clinical Competency in AM in general and notably in internal medicine where the same was true for Clinical Competencies in CON. Possible reasons for the better performance of Swiss hospitals lie partly in their better structural department features aimed at a systematic training of AM in PGMT. However, the better performance of Swiss hospitals in other dimensions such as Global Satisfaction lost statistical significance when department size was accounted for. In German hospitals with their larger clinical departments, department size was negatively correlated with quality of PGMT as well as with the working situation. Apart from department size, the latter may also be influenced by systemic problems connected with increased workload, financial and time pressure such as the DRG system which was only operative in Germany. Consequently, the ability to complete continuing education during working hours in departments of comparable size was significantly better in Switzerland. Thus, our results suggest that structural, systemic (especially economic) and possibly other reasons may be responsible for the suboptimal quality of AM and CON aspects in integrative PGMT. For this reason, we will publish two additional papers containing a thorough analysis of the obstacles to an optimal integrative PGMT with AM as well as possible solutions to these problems [31,34].

## Abbreviations

AM: Anthroposophic medicine; CAM: Complementary and alternative medicine; CH: Switzerland (Confoederatio Helvetica); CON: Conventional medicine; D: Germany; DRG: Disease related groups; IM: Integrative medicine; PGMT: Postgraduate medical training; TE: Trainees; TR: Trainers.

## Competing interests

None of the authors have financial or non-financial competing interests.

## Authors’ contributions

PH and SE conceived the overall study idea and developed the research design jointly with PO. PO provided the questionnaire for CON aspects of PGMT and performed the statistical evaluation, SE and PH developed the module for AM aspects of PGMT along with PO. SE and PO organized and carried out the survey. PH drafted the content and JW provided the illustrations and formal aspects of the manuscript. SE, PO, JW and BB contributed to the final form of the manuscript. All authors read and approved the final manuscript.

## Pre-publication history

The pre-publication history for this paper can be accessed here:

http://www.biomedcentral.com/1472-6882/14/191/prepub
